# Integrated strategy of network analysis prediction and experimental validation to elucidate the possible mechanism of compound Turkish gall ointment in treating eczema

**DOI:** 10.1186/s13020-022-00643-2

**Published:** 2022-07-30

**Authors:** Xuan Ma, Meng Hao, Ming Hui Zhang, Ya Zeng, Qing Qing Yang, Lu Zhao, Chen Yang Fan, Zhi Hong Ji, Ke Ao Li, Zhi Jian Li, Mirzat Maimaiti, Ji Hong Nie

**Affiliations:** 1 College of Pharmacy, Xinjiang Medical University, Xinjiang, Urumqi, 830011 China; 2 Xinjiang Qimu Medical Research Institute, Xinjiang, Urumqi, 830002 China; 3 Xinjiang Key Laboratory of Generic Technology of Traditional Chinese Medicine (Ethnic Medicine) Pharmacy, Xinjiang, Urumqi, 830002 China; 4 Xinjiang Uygur Medical Research Institute, Xinjiang, Urumqi, 830011 China; 5 Department of Pharmacy, Affiliated Hospital of Traditional Chinese Medicine, Xinjiang Medical University, Xinjiang, Urumqi, 830011 China; 6 Xinjiang Key Laboratory of Processing and Research of Traditional Chinese Medicine, Xinjiang, Urumqi, 830011 China

**Keywords:** Eczema, TLR4/NF-κB signaling pathway, CTGO, HPLC fingerprint, Network analysis

## Abstract

**Background:**

Compound Turkish gall ointment (CTGO) has a long history of being widely used as a folk medicine in Xinjiang for the treatment of eczema. CTGO is currently in the pre-investigational new drug application stage, but its pharmacological mechanisms of action have not yet been clarified.

**Methods:**

First, a sensitive and reliable ultra-high performance liquid chromatography-Q exactive hybrid quadrupole-orbitrap high-resolution accurate mass spectrometry (UHPLC-Q-Orbitrap HRMS) technique was established. Second, an integrative strategy of network analysis and molecular docking based on identified and retrieved ingredients was implemented to investigate the potential targets and pathways involved in the treatment of eczema with CTGO. Finally, Sprague–Dawley (SD) rats with eczema were prepared to verify the predicted results. The skin conditions of the rats were observed, evaluated, and scored. Skin tissues were observed by hematoxylin–eosin (HE) staining, and the levels of serum interferon-γ (IFN-γ) and interleukin-4 (IL-4) were determined by enzyme-linked immunosorbent assay (ELISA). The expression levels of toll-like receptor 4 (TLR4), nuclear factor kappa-B p65 (NF-κB p65), interleukin-1β (IL-1β), and tumor necrosis factor-α (TNF-α) were detected by real-time quantitative polymerase chain reaction (RT-qPCR).

**Results:**

A total of 29 compounds were identified. We found 38 active components and 58 targets for the treatment of eczema, which included 118 signaling pathways related to inflammation, immunity, and apoptosis. CTGO significantly improved the skin surface and histopathological characteristics of eczema-affected rats, downregulated the expression of IL-4, TLR4, NF-κB (p65), IL-1β, and TNF-α, and upregulated the expression level of IFN-γ.

**Conclusion:**

We predicted and validated our prediction that CTGO may be used to treat eczema by affecting the TLR4/NF-κB signaling pathway, which provides guidance for future experimental studies.

## Background

Eczema is a chronic inflammatory skin disease with exudative tendency and clinical manifestations including erythema, blisters, erosion, and pruritus [1]. Modern medicine believes that the main etiology of eczema is T cell-mediated immune damage. Antihistamines and glucocorticoid drugs are often used for clinical treatment of eczema; however, these drugs often lead to adverse reactions and frequent recurrence, and also have a high cost [2, 3]. Traditional Chinese medicine (TCM) has a long history in the treatment of eczema. Three pathogenic factors of rheumatic fever are considered the main causes of eczema. Treatment mainly focuses on removing wind and dampness, cooling blood, and detoxification [4–6]. Network analysis is based on a “drug-composition-target-disease” relationship characterized by comprehensive analysis, and has been introduced to evaluate the constituents and action mechanism of TCM in view of its systematic and holistic coincidences [7–9]. CTGO comprises Turkish gall and Comfrey. Turkish gall, the gall of Quercus infectoria Oliv, is a traditional Uygur medicine distributed in the Mediterranean coast, Arabia, Turkey, Greece, and Iran. The main components of CTGO are tannin and gallic acid, which have anti-inflammatory, drying, astringent, and hemostatic effects [10, 11]. Comfrey is the dried root of Arnebia euchroma, and is distributed in Xinjiang, Tibet, Gansu, and other places. Comfrey comprises numerous naphthoquinones and polysaccharides, and has anti-bacterial, anti-inflammatory, and immunological regulatory effects [12]. In TCM, comfrey has the effects of dryness and dampness astringency, clearing heat and cooling blood, and detoxification. In long-term clinical application, CTGO has been shown to have a good effect on eczema. Based on the network analysis of the potential components, targets, and mechanisms of CTGO in the treatment of eczema, we explored the therapeutic effect and mechanism of CTGO in a rat model of eczema to provide a theoretical basis for the treatment of eczema with CTGO.

**Fig. 1:**
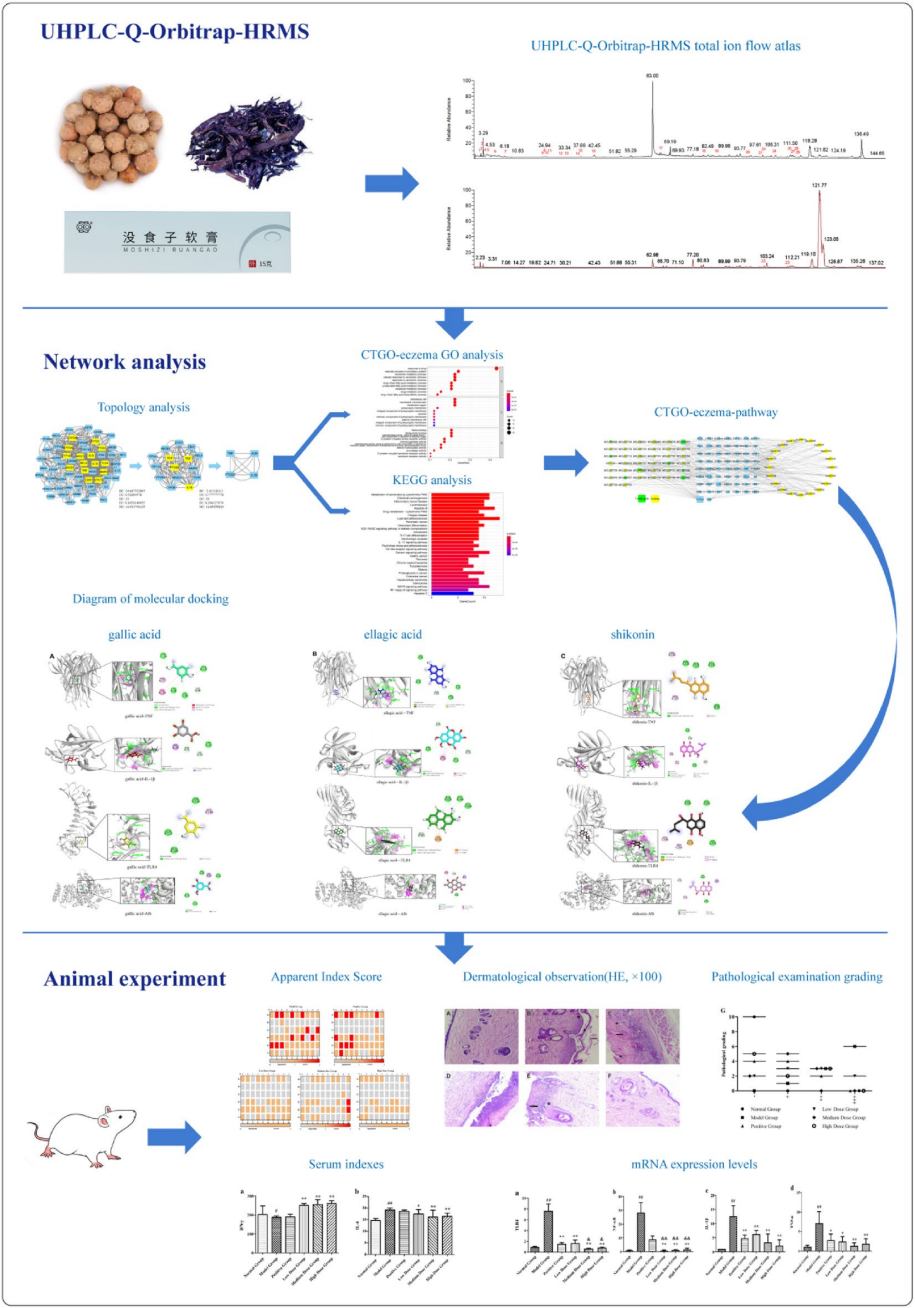
The flow chart of the study

We systematically expounded the possible targets and related pathways of CTGO in treating eczema by integrating UHPLC-Q-Orbitrap HRMS, network analysis, molecular docking analysis, and experimental evaluation using molecular biology. Figure 1 shows the flow chart of the analysis.

## Materials

### Materials and equipment

Low (0.035 g/g, batch No. 20200506), medium (0.07 g/g, batch No. 200401), and high (0.14 g/g, batch No.20200507) dose CTGO were purchased from NEW CICON Pharmaceutical Co., Ltd.; Gallic acid (content, 91.5%, Batch No. 110831–201906), Ellagic acid (content: 88.8%, Batch No. 111959–201903), and β,β-Dimethylacrylalkannin (content: 98.0%, Batch No. 111689–201805) were purchased from China Institute for Food and Drug Control; Gallic acid methyl ester (content: 98.0%, Batch No. 17092604) and Ethyl gallate (content: 98.0%, Batch No. 19042902) were purchased from Chengdu Pufei De Biotech Co., Ltd.; L-shikonin (Batch No. 110769–200506) was purchased from China Institute for the Control of Pharmaceutical and Biological Products; Methanol and acetonitrile of HPLC grade were purchased from Sigma-Aldrich Company. All other reagents were of analytical grade and supplied by Tianjin Beilian Fine Chemicals Development Co., Ltd. Compound dexamethasone acetate cream (20201024X) was purchased from China Resources Sanjiu Medical and Pharmaceutical Co., Ltd.; 2, 4-dinitrochlorobenzene (DNCB, 97–00-7) was purchased from Shanghai Jiudin Chemical Technology Co., Ltd.; IL-4 and INF-γ test box (05/2020, 06/2020) were purchased from Shanghai Enzyme Linked Biotechnology Co., Ltd.; the PCR kit (AQ601-01) was purchased from Beijing Quanshijin; the microscope (EQ2016-037) was purchased from Leica, Germany; the Multiskan FC was purchased from Thermo Fisher; and the electronic balance (CPA124S) was purchased from Sartorius, Germany.

### Experimental animals

Sixty Sprague–Dawley (SD) rats (180–220 g body weight, aged 5–7 weeks) were purchased from the Laboratory Animal Center of Xinjiang Medical University (License number: SCXK (new) 2018–0002). All of the rats were fed in an environment free of specific pathogens, at a temperature of 20–26 °C, humidity 40%– 70%, artificial light, 12-h light/dark cycle, and had free access to food and water.

### Database and analysis software

The following databases were used: TCMSP (http://tcmspw.com/index.php/), CNKI (https://www.cnki.net/), CTD (http://ctdbase.org/), ETCM (http://www.tcmip.cn/ETCM/index.php/Home/Index/

), TCMID (http://47.100.169.139:8000/tcmid/), BATMA.

N-TCM (http://bionet.ncpsb.org.cn/batman-tcm/), GEO (https://www.ncbi.nlm.nih.g

ov/geo/), DrugBank (https://go.drugbank.com/), STRING (https://string-db.org/), Pu.

bChem (https://pubchem.ncbi.nlm.nih.gov/), and PDB (http://www1.rcsb.org/). The software Cytoscape 3.7.2, R3.6.1 language, Chem3D, PyMOL, AutoDock Vina-1.5.6, and BIOVIA Discovery Studio 2020 were used.

### Untargeted UHPLC-Q-Orbitrap MS analysis

Chromatographic separation was performed on a Q-Exactive-Plus (Thermo Fisher Scientific Inc., Waltham, MA, USA) using a Kromasil Classic 100–5-C18 column (100 × 4.6 mm i.d., 5 μm) at 30 °C, with a flow rate of 1.0 mL/min and a wavelength of 300 nm. The mobile phase consisted of water containing 0.1% formic acid (solvent system A) and acetonitrile (solvent system B). Gradient elution was conducted as follows: 0–5 min, 5% B; 5–45 min, 5–12% B; 45–50 min, 12% B; 50–80 min, 12–20% B; 80–95 min, 20–25% B; 95–105 min, 25–67% B; 105–115 min, 67–70% B; 115–125 min, 70–75% B; 125–135 min, 75% B. Ten microliters of the sample solution was injected for analysis.

Mass detection was performed using an UHPLC-Q-Orbitrap HRMS equipped with a Dual ESI source operating in both positive and negative modes with the following operating parameters: ESI spray voltage, –2.8 kV (positive ion, 3.2 kV); sheath gas, 40 arb; auxiliary gas, 10 arb; air curtain gas (CUR), 35; ion source temperature (heat temp), 350 °C; capillary temperature, 300 °C; –70 V; focusing voltage (FP), –350 V; and DP2, –10 V. The data dependent acquisition (DDA) mode was adopted for sample analysis; the collection range of the q-orbitrap was 100–1500 m/z, and the fragment ion scanning range was 50–1500 m/z. The collision gas (NCE): 20, 40, 60. The mass spectrometry (MS) resolution was 70,000 full width at half maximum (FWHM) (200 m/z), and the MS^2^ resolution was 17,600 FWHM (200 m/z). The Xcalibur 4.0 software package was used for data acquisition and analysis (Thermo Fisher Scientific Inc., Waltham, MA, USA).

To prepare the sample, 0.05 g of Turkish gall was weighed and placed into a conical flask. Next, 25 mL of 80% methanol was added before a 1-h reflux in a water bath at 75 °C. Then, a pipette was used to transfer 1 mL of the supernatant into a 5 mL volumetric flask. Simultaneously, 0.1 g of Comfrey was weighed precisely and placed into another conical flask, before adding 50 mL of chloroform and subjecting it to a 1 h reflux in a water bath at 75 °C. The sample was then filtered, and the solvent was evaporated in the water bath. The residue was transferred into the abovementioned 5-mL volumetric flask with approximately 4 mL of acetonitrile to bring the total volume to 5 mL.

### Network analysis

#### Target gene collection

“Turkish gall” and “Comfrey” were searched for in the TCMSP, CTD, TCMID, ETCM, and BATMAN-TCM databases and the literature to obtain the components of Turkish gall and Comfrey. These agents were combined with the compounds identified by UHPLC-Q-Orbitrap-HRMS to build the active ingredient database of CTGO. Targets corresponding to the components were obtained through the TCMSP and BATMAN-TCM databases, and a target database of CTGO was constructed. “Eczema” was searched in the GEO and DrugBank databases to obtain eczema targets. R 3.6.1 language software was used to obtain the intersection targets of drugs and diseases.

#### Protein–protein interaction (PPI) network analysis

To clarify the relationship between intersection targets, an intersection target PPI network of eczema and CTGO components was constructed and analyzed using the STRING database. Species were defined as Homo sapiens. PPIs with a confidence score > 0.40 were selected and topology analyses were conducted to ensure the accuracy of the results. The PPI network was imported into the Cytoscape 3.7.2 software for use with PPI topology analysis.

#### GO and KEGG enrichment analysis

GO enrichment analysis was performed on the intersection targets of CTGO with eczema using R language software. According to the number and significance of gene enrichment (*q* < 0.05), the top 10 GO biological processes, cellular composition, and molecular functions were selected to draw bubble maps; the intersection targets of CTGO and eczema were analyzed by KEGG enrichment. According to the number and significance of gene enrichment (*q* < 0.05), the top 20 pathways were selected and bar graphs were drawn. The size of the bubbles and strips in the figure represents the number of enriched genes on this pathway, and the color difference of the bubbles and strips represents the level of enrichment of target genes on this pathway.

#### Network of component-target-pathway

The intersection targets and corresponding components and the top 20 pathways of significance were input to Cytoscape 3.7.2 software to construct a “component-target-pathway” network, and the size of degree value was reflected by the size of the nodes.

#### Molecular docking

The three core components of gallic acid, ellagic acid, and shikonin in the component-target-pathway network were docked with the four core targets of TNF, IL-1β, TLR4, and albumin (Alb) in the PPI network. First, the composition and target files were downloaded from PubChem and PDB. Next, energy minimization of the component file was completed by Chem3D, and dewatering and impurity removal of the target file were completed by PyMOL. The file was then converted to PDBQT format using AutoDock Vina. The active pocket of the target was determined, molecular docking was performed, and binding energy values were obtained. The best docking model diagram was drawn by BIOVIA Discovery Studio. Finally, the binding ability was evaluated by the affinity of the component and the target.

### Animal experiment

#### Grouping and modeling

Sixty rats were randomly divided into a normal group, a model group, a positive group (compound dexamethasone acetate cream), a CTGO high-dose group, a CTGO medium-dose group, and a CTGO low-dose group, with 10 rats per group. The rats in all groups, except for the normal group, were treated with 7% DNCB solution by rubbing the solution on the back to establish the eczema model. The hair on the neck (A: 2 cm × 2 cm) and back (B: 4 cm × 4 cm) of the animals was removed using an electric shaver. The rats were sensitized by applying 100 μL of 7% DNCB acetone solution with a pipetting gun on the skin of site A. Severe pruritus, frequent scratching, and rolling behaviors were observed in rats, lasting for approximately 2 h. After 1 week, 200 μL of 7% DNCB acetone solution was applied to site B for excitation. The rats were stimulated once every 5 days, and in each stimulation, severe pruritus could be seen in rats, with frequent scratching and rolling behavior. Erythema, papules, edema, scratches, and desquamation gradually appeared on the skin at site B. After each stimulation, the skin lesions were recorded and scored. After six sessions of stimulation, erythema, papules, scab, and exudation appeared in skin lesions on the back of each rat, at which point the stimulation was stopped, indicating that the model had been successfully established (Miao MS, et al., 2017).

#### Drug treatments

Each group was administered a low (0.035 g/g), medium (0.07 g/g), or high (0.14 g/g) dose of CTGO, and the positive group was administered compound dexamethasone acetate cream (0.75 mg/g, 0.3 g each time, once a day). The normal and model groups were administered with saline. After smearing the experimental drug, 2–3 layers of medical gauze were wrapped and fixed with a non-irritating adhesive tape. After 4 h, the covering was removed and washed with warm pure water for continuous administration for 14 days.

#### Sample collection

After the final administration, the rats were fasted with water for 12 h. The rats were mildly anesthetized with 1% sodium pentobarbital (4 mL/kg, i.p.). Blood samples were collected from the abdominal aorta and placed in a centrifuge tube for 30 min at 3000 rpm at 4 °C, before storing the supernatant at –20 °C. One part of the obtained skin tissue was immersed in 10% neutral formalin for fixation, and the other part was stored at − 80 °C.

### Detection item

#### Observation of apparent indicators

On days 1, 5, 10, 15, 20, 25, 30, and 35 (administration D0) of modeling, and on days 0, 9, and 14 of administration, the grades of erythema, edema, exudation, desquamation, liche-like change, desquamation were scored from 0 to 2. A score of 0 represents no symptoms; 1 represents mild symptoms; and 2 represents significant symptoms After 14 days of administration, the skin conditions were photographed observed with the naked eye.

#### HE staining

Skin tissue was removed from 10% neutral formalin solution, dehydrated, permeated, paraffin impregnated, embedded, sectioned, stained, and sealed. Histomorphological observation was performed with a microscope, and radiographs were taken.

#### ELISA

The levels of IFN-γ and IL-4 in the serum of rats were determined according to the instructions of the ELISA kit.

#### RT-qPCR

The mRNA expression levels of TLR4, NF-κB (p65), IL-1β, and TNF-α in skin tissue samples were detected by RT-qPCR. RNA was extracted, its purity was determined, and cDNA was obtained by reverse transcription. PCR upstream and downstream primers were added (2 µL each; refer to Table 1 for specific information) to the PCR reaction system of 20 µL according to the kit instructions. The reaction procedures were as follows: pre-denaturation at 95 °C for 5 min, denaturation at 95 °C for 5 s, and annealing at 60 °C for 35 s; detection was conducted after 45 cycles. The 2^−△△CT^ method was used to analyze the difference in target gene expression between the control group and test groups.

**Table 1:** PCR primer sequences

Gene	Sequence
β-actin	F-CCCATCTATGAGGGTTACGC
	R-TTTAATGTCACGCACGATTTC
TLR4	F-TCCTTTCCTGCCTGAGACCA
	R-TGTCTCAATTTCACACCTGGAT
NF-κB	F-TGTATTTCACGGGACCTGGC
	R-CAGGCTAGGGTCAGCGTATG
IL-1β	F-AAATGCCTCGTGCTGTCTGA
	R-TTGGGATCCACACTCTCCAG
TNF-α	F-GTCCCAACAAGGAGGAGAAGTT
	R-CTCCGCTTGGTGGTTTGCTA

### Statistical analysis

All data are presented as the mean ± standard deviation. All statistical analyses were conducted using SPSS software, version 23.0. Multiple groups of measurement data were analyzed by ANOVA, and multiple comparisons were analyzed by LSD. *P* < 0.05 was considered statistically significant.

## Results

### Results of UHPLC-Q-Orbitrap-HRMS

**Fig. 2:**
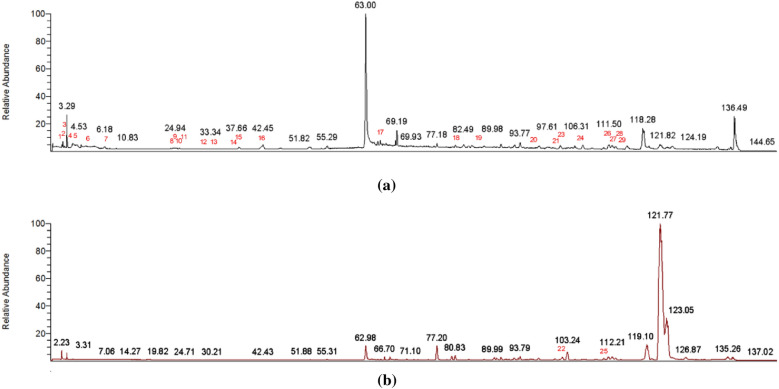
UHPLC-Q-Orbitrap-HRMS total ion flow atlas. **a** Total ion current diagram in negative ion mode; **b** total ion current diagram in positive ion mode

All of the fragment ions were analyzed and identified, and a total of 29 compounds were identified by referring to the literature and comparing with existing databases. The chemical formulae of compounds 2 and 3 were both C_13_H_16_O_10_, which were preliminarily identified as galloyl-glucoside. The chemical formulae of compounds 6, 7, 8, and 10 were all C_20_H_20_O_14_, which were preliminarily identified as galloyl-glucoside. The chemical formulas of compounds 11, 12, 13, and 14 were all C_27_H_24_O_18_, which were preliminarily identified as trigalloyl-glucoside. The above compounds were all C_27_H_24_O_18_. Although the chemical formula is the same, there may be differences in the position at which the groups are connected, and the specific chemical structural formula needs to be further determined by methods such as nuclear magnetic resonance scanning. In addition to the above 10 compounds, 19 compounds were also analyzed and identified, including 4 phenolic acid compounds, 3 ester compounds, 11 naphthoquinone compounds, and 11 tannin compounds. The specific experimental results are shown in Fig. 2 and Table 2.

**Table 2:** Results of UHPLC-Q-Orbitrap-HRMS analysis

No	tR (min)	[M-H]^−^	[M + H]^+^	Error (ppm)	Formula	MS/MS	Identification
1	2.48	191.05595		4.9171	C_7_H_12_O_6_	173 (5), 127 (10), 93 (20), 85 (40)	Quinic acid
2	2.79	331.06683		2.5990	C_13_H_16_O_10_	271 (10), 211 (50), 169 (100), 125 (60)	Galloyl-glucoside
3	3.20	331.06680		2.5068	C_13_H_16_O_10_	271 (20), 211 (30), 169 (100), 125 (60)	Galloyl-glucoside
4	3.36	481.06274		3.0404	C_20_H_18_O_14_	331(10), 313 (5), 301 (100), 275 (60),	HHDP-glucoside
5	4.87	169.01407		4.9285	C_6_H_12_O_7_	257 (30), 169 (40), 125 (30)	Gallic acid
6	7.44	483.07849		3.2285	C_20_H_20_O_14_	125 (100)	Di-galloyl-glucoside
7	10.90	483.07962		3.2285	C_20_H_20_O_14_	313 (20), 271 (5), 211 (5), 169 (100), 125 (70)	Di-galloyl-glucoside
8	23.98	483.07837		2.9785	C_20_H_20_O_14_	313 (20), 271 (5), 211 (5), 169 (100), 125 (70)	Di-galloyl-glucoside
9	24.94	183.02925		2.4656	C_8_H_8_O_5_	331 (10), 313 (20), 271 (30), 211 (80), 169 (100), 25 (70)	Methylgallate
10	25.42	483.07858		3.4181	C_20_H_20_O_14_	169 (20), 125 (30)	Di-galloyl-glucoside
11	26.93	635.08850		0.9618	C_27_H_24_O_18_	331 (10), 313 (20), 271 (70), 211 (70), 169 (100), 125 (60)	Tri-galloyl-glucoside
12	29.54	635.08960		2.6917	C_27_H_24_O_18_	483 (20), 465 (60), 313 (30), 169 (100), 125 (70)	Tri-galloyl-glucoside
13	32.91	635.0899		3.8451	C_27_H_24_O_18_	483 (20), 465 (40), 313 (32), 169 (100), 125 (80)	Tri-galloyl-glucoside
14	35.79	635.09015		3.9612	C_27_H_24_O_18_	483 (20), 465 (80), 313 (30), 169 (100), 125 (80)	Tri-galloyl-glucoside
15	37.56	197.04541		4.8734	C_9_H_10_O_5_	169 (50), 125 (40)	Ethyl gallate
16	42.42	300.99780		–0.3017	C_14_H_6_O_8_	257 (10), 229 (10), 185 (5)	Ellagic acid
17	66.51	165.05573		6.6912	C_9_H_10_O_3_	137 (80), 108 (20), 93 (80	Methyl p-hydroxybenzoate
18	80.22	287.09277		4.7837	C_16_H_16_O_5_	218 (100), 190 (20), 173 (5)	L-shikonin
19	84.29	627.2245		3.3077	C_36_H_36_O_10_	567 (80), 507 (100), 463 (50), 410 (20), 347 (10), 284 (10)	6-(11′-Deoxyalkannin)-alkannin /shikonin isobutyrylate
20	97.44	387.14536		3.9409	C_21_H_24_O_7_	285 (5), 269 (70), 251 (60), 225 (30), 186 (20), 117 (100)	β-Hydroxy isovaleryl Shikonin
21	100.55	329.10321		3.7852	C_18_H_18_O_6_	269 (100), 251 (90), 225 (40), 186 (50)	Acetylshikonin
22	101.65		271.0963	–0.5217	C_16_H_16_O_4_	253 (60), 243 (20), 229 (90), 225 (40), 137 (70), 93 (20)	Shikonin
23	101.69	429.15564		2.9022	C_23_H_26_O_8_	369 (5), 269 (100), 251 (40), 225 (30), 186 (20)	β-Acetoxy isoamyl acarnin
24	106.18	357.13461		3.7750	C_20_H_22_O_6_	269 (100), 251 (70), 225 (60), 186 (30)	Isobutyryl Shikonin
25	110.49		271.0963	–0.5217	C_16_H_14_O_4_	253 (60), 229 (100), 165 (20), 137 (80)	Deoxyshikonin
26	110.51	369.13449		3.3216	C_21_H_22_O_6_	269 (100), 251 (80), 225 (50), 186 (40)	β,β′-Dimethyl acryloyl Shikonin
27	111.596	455.35361		3.5914	C_30_H_48_O_3_	153 (5), 113 (10), 69 (30)	Ursolic acid
28	112.85	269.08228	271.09647	–0.0715	C_16_H_14_O_4_	253 (60), 229 (100), 165 (30), 137 (70)	Deoxyshikonin-isomer
29	112.90	371.15012		3.2361	C_21_H_24_O_6_	269 (100), 251 (70), 225 (30), 186 (40)	α-Methyl n-butyryl shikonin/ isovaleryl Shikonin

### Network pharmacological analysis

#### Targets of CTGO in the treatment of eczema

The results of CTGO active ingredient and target screening showed that 59 CTGO chemical components were retrieved from the database and literature; 8 from Turkish gall, 51 from Comfrey, and 57 from deduplication, as shown in Table 3. The 57 components obtained by retrieval were combined with the 29 components obtained by mass spectrometry, among which 9 components were overlapped, totaling 77 active components after de-repetition. Additionally, 886 targets of 77 components of CTGO were obtained from the TCMSP and BATMAN-TCM databases, and 193 eczema targets were obtained through the GEO and DrugBank databases. The intersection targets of 58 CTGO and eczema were obtained by analysis using R language software.

**Table 3:** “Component-target” active ingredient

No	Traditional Chinese medicine	MolId	MolName	No. of eczema-related targets	Ingredient source
1	Turkish gall	MOL000513	Gallic acid	21	A and B
2	Turkish gall/Comfrey	MOL000069	Palmitic acid	17	B
3	Turkish gall	MOL001002	Ellagic acid	17	A and B
4	Turkish gall	MOL002037	Amentoflavone	4	B
5	Turkish gall	MOL001907	Progallin A/Ethyl gallate	3	A and B
6	Turkish gall/Comfrey	MOL000359	Sitosterol	3	B
7	Turkish gall	MOL001906	Methylgallate	2	A and B
8	Turkish gall	MOL000569	Digallate	1	B
9	Comfrey	MOL000131	EIC	15	B
10	Comfrey	MOL001492	Ethyl icosanoate	/	B
11	Comfrey	MOL001493	Ethyl margarate	/	B
12	Comfrey	MOL001494	Mandenol	3	B
13	Comfrey	MOL001498	Ethyl stearate	/	B
14	Comfrey	MOL001499	Ethyl tetracosanate	/	B
15	Comfrey	MOL000223	Caffeic acid	23	B
16	Comfrey	MOL002372	(6Z,10E,14E,18E)-2,6,10,15,19,23-hexamethyltetracosa-2,6,10,14,18,22-hexaene	/	B
17	Comfrey	MOL000263	Oleanolic acid	6	B
18	Comfrey	MOL002691	Iva	25	B
19	Comfrey	MOL002883	Ethyl oleate (NF)	1	B
20	Comfrey	MOL003616	Isobutyryl shikonin	16	A and B
21	Comfrey	MOL003619	[(1R)-1-(5,8-dihydroxy-1,4-diOxo-2-naphthyl)-4-methyl-pent-3-enyl] 3-methylbutanoate	10	B
22	Comfrey	MOL004784	Stenol	/	B
23	Comfrey	MOL000511	Ursolic acid	55	A and B
24	Comfrey	MOL005705	Eicosanol	/	B
25	Comfrey	MOL005796	Lignocerol	/	B
26	Comfrey	MOL005855	Tormentic acid	1	B
27	Comfrey	MOL000663	Lignoceric acid	2	B
28	Comfrey	MOL000675	Oleic acid	48	B
29	Comfrey	MOL007060	Lithospermic acid B	/	B
30	Comfrey	MOL007714	1-methoxyacetylshikonin	14	B
31	Comfrey	MOL007715	[(1R)-1-(5,8-dihydroxy-1,4-diOxo-2-naphthyl)-4-methyl-pent-3-enyl] propanoate	16	B
32	Comfrey	MOL007716	Acetylshikonin	19	A and B
33	Comfrey	MOL007717	Alkannin beta,beta-dimethylacrylate	12	B
34	gongComfrey	MOL007718	Methyl nervonate	/	B
35	Comfrey	MOL007719	Arnebin 7/Deoxyshikonin	15	A and B
36	Comfrey	MOL007720	(2R)-3-oxo-2-phenylbutanenitrile	18	B
37	Comfrey	MOL007721	Totarol	/	B
38	Comfrey	MOL007722	Isoarnebin 4	7	B
39	Comfrey	MOL007723	Alkannan	12	B
40	Comfrey	MOL007724	Senecic acid	12	B
41	Comfrey	MOL007725	5-Methylphthalide	/	B
42	Comfrey	MOL007726	Ethyl docosanoate	/	B
43	Comfrey	MOL007727	Ethyl senecioate	1	B
44	Comfrey	MOL007728	Lithospermidin A	9	B
45	Comfrey	MOL007729	Shikonofuran C	4	B
46	Comfrey	MOL007730	Shikonofuran B	5	B
47	Comfrey	MOL007731	Arnebinol	12	B
48	Comfrey	MOL007732	Arnebinone	8	B
49	Comfrey	MOL007733	Butyric acid, 3-hydroxy-3-methyl-	/	B
50	Comfrey	MOL007734	5-[(E)-5-(3-furyl)-2-methyl-pent-2-enyl]-2,3-dimethoxy-p-benzoquinone	5	B
51	Comfrey	MOL007735	Des-O-methyllasiodiplodin	/	B
52	Comfrey	MOL007736	Lithospermidin B	6	B
53	Comfrey	MOL007737	Alpha-methyl-n-butylshikonin	13	A and B
54	Comfrey	MOL007738	Beta-acetoxyisovalerylshikonin	6	B
55	Comfrey	MOL007739	[(1R)-1-(5,8-dihydroxy-1,4-dioxo-2-naphthyl)-4-methyl-pent-3-enyl] 3-hydroxy-3-methyl-butanoate	/	B
56	Comfrey	MOL007740	[(1R)-1-(5,8-dihydroxy-1,4-dioxo-2-naphthyl)-4-methyl-pent-3-enyl] 2-methylpropanoate	25	B
57	Comfrey	MOL000861	Healip	/	B

A represents the components detected by mass spectrometry, B represents the components obtained from the database and literature search

#### PPI network construction and topology analysis

**Fig. 3:**
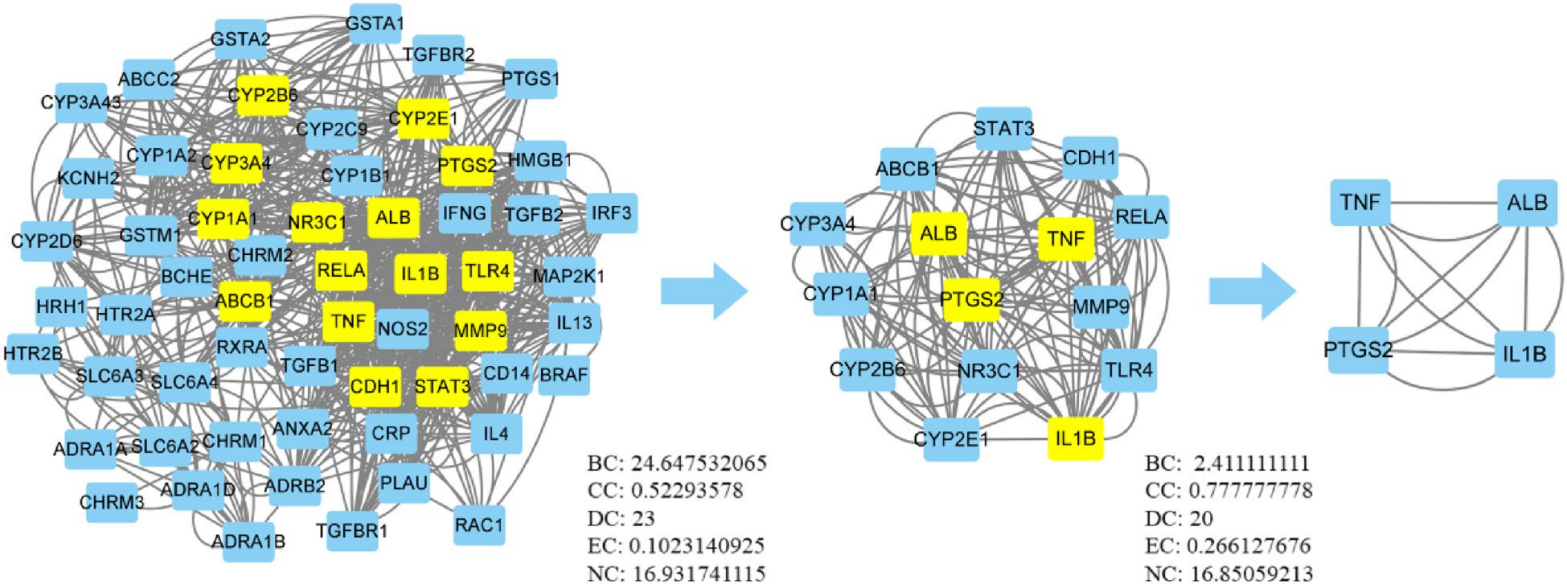
PPI topology analysis. Targets are indicated by rectangles. The nodes representing candidate compounds are shown as yellow rectangles

PPI topology analysis results showed that after importing the intersection targets into the STRING data platform, the PPI relationship between the intersection targets was obtained. The PPI analysis network was obtained using Cytoscape 3.7.2 for topology analysis of the relationship. The network consisted of 58 nodes and 390 edges. The average node degree value was 13.4, and the PPI enrichment *P*-value was < 1.0 × 10^−16^. The top four targets in degree value included Alb, tumor necrosis factor (TNF), IL1β, and TLR4. In PPI topology analysis, the six topological eigenvalues of “closeness centrality (CC),” “betweenness centrality (BC),” “eigenvector centrality (EC),” “local average connectivity (LAC),” “degree centrality (DC),” and “network centrality (NC)” were used as filter conditions. Targets with topological eigenvalues greater than their corresponding medians were used as core targets, and two rounds of analysis were performed. Fifteen core targets were obtained in the first round and four were obtained in the second round, as shown in Fig. 3.

#### GO and KEGG enrichment analyses

**Fig. 4:**
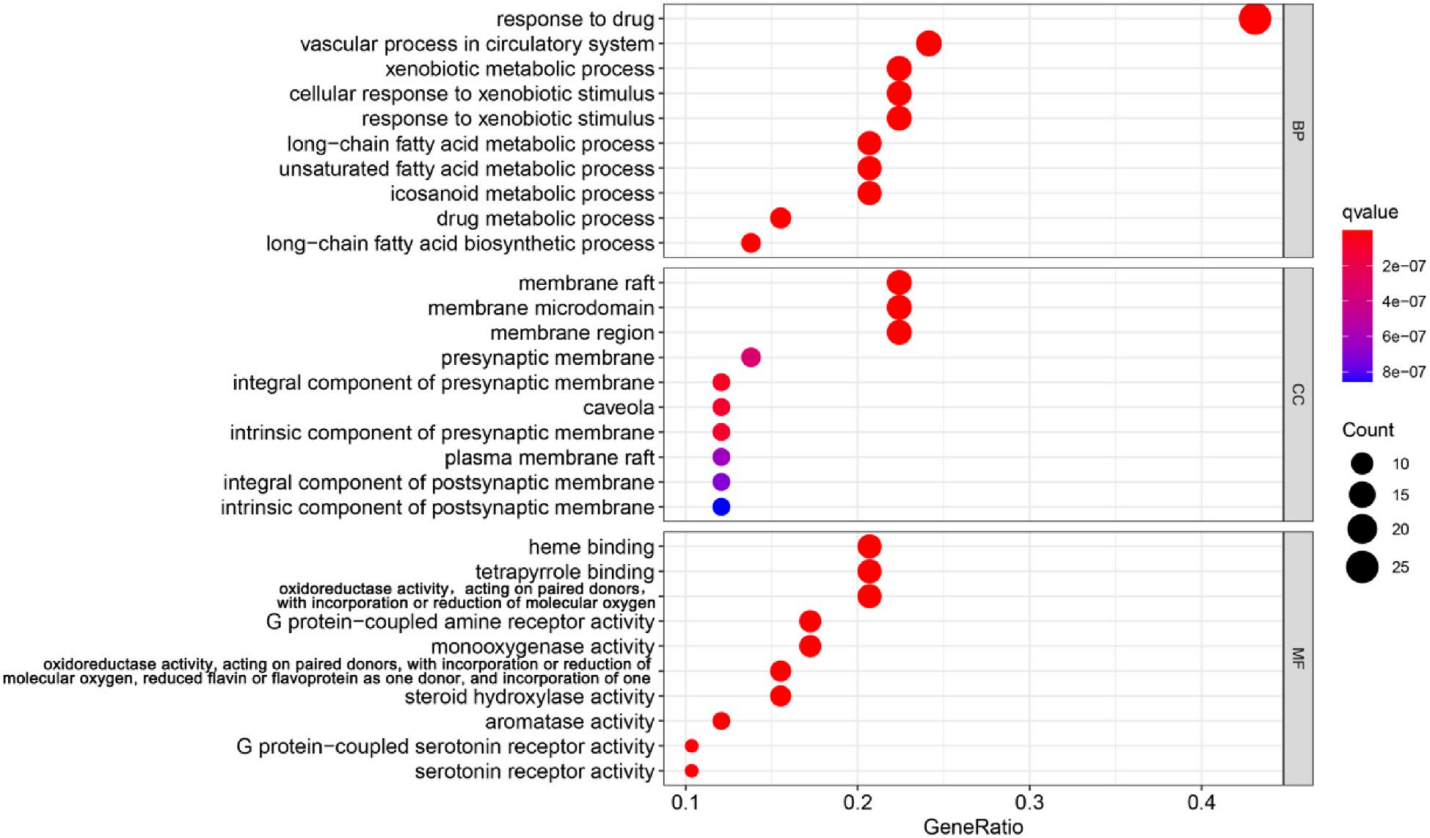
CTGO-eczema GO analysis. GO items with significant changes (*q* < 0.05) were identified. The vertical coordinates represent the GO item with significant enrichment, and the horizontal coordinates represent the ratio of differentially expressed genes in each item. The color of the bubble indicates the significance of the enriched GO item, and the color gradient represents the size of the *q*-value. The size of the bubble represents the number of genes

**Fig. 5:**
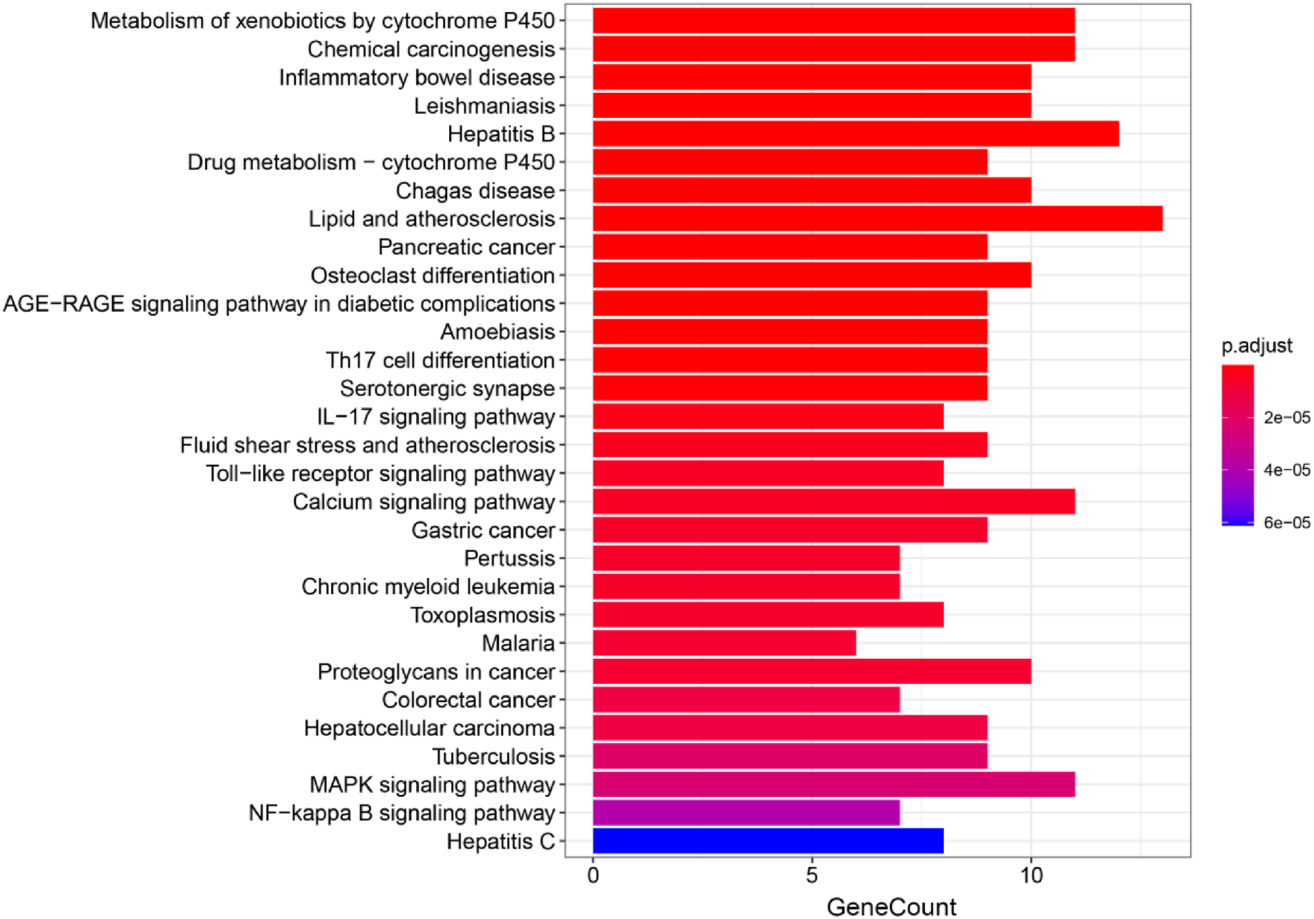
CTGO-eczema KEGG analysis. Top 30 KEGG pathway enrichment candidate targets for CTGO activity against eczema for each expression profile. Pathways with significant changes (*q* < 0.05) were identified. The vertical coordinates represent the KEGG pathway with significant enrichment, and the horizontal coordinates represent the number of differentially expressed genes in each pathway. The color of the bar graph indicates the significance of the enriched KEGG pathway, and the color gradient represents the size of the *q*-value

GO and KEGG enrichment analyses and visualization processing were performed on 58 intersections of CTGO and eczema using the Bioconductor bioinformatics software package. GO functional annotation showed that 1420 (*q* < 0.05) GO functional enrichment items were obtained, including 1316 biological processes (BPs), 75 molecular functions (MFs), and 29 cellular components (CCs), as shown in Fig. 4. KEGG pathway enrichment analysis showed that 118 (*q* < 0.05) signaling pathways were obtained, as shown in Fig. 5.

#### CTGO-eczema “component-target-pathway” network construction

**Fig. 6:**
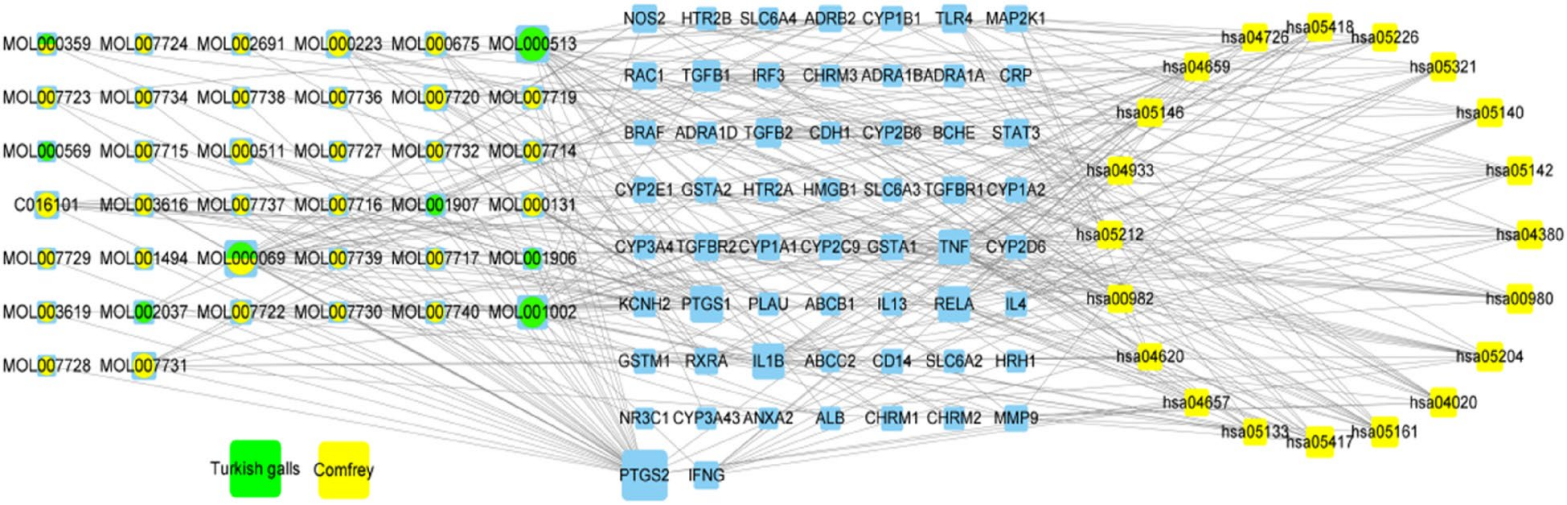
CTGO-eczema “component-target-pathway” network. Blue rectangles represent the target, circles represent a common compound, yellow squares represent the pathway. The node size and the degree show a positive proportional relationship

The “component-target-pathway” network showed that after matching 58 intersection targets with 77 active ingredients, 38 active ingredients with a therapeutic effect on eczema were finally obtained. A total of 38 active ingredients and 58 intersection targets and the top 20 pathways of significance were imported into Cytoscape 3.7.2 software to construct the “component-target-pathway” regulation network of TCM prescriptions. The top four core components in the ranking of degree value were gallic acid, palmitic acid, ellagic acid, and shikonin. The top 10 degree value core targets were RELA, TNF, IL1B, TGFB1, TGFB2, TLR4, IFNG, TGFBR2, TGFBR1, and STAT3, as shown in Fig. 6.

#### Molecular docking

**Fig. 7:**
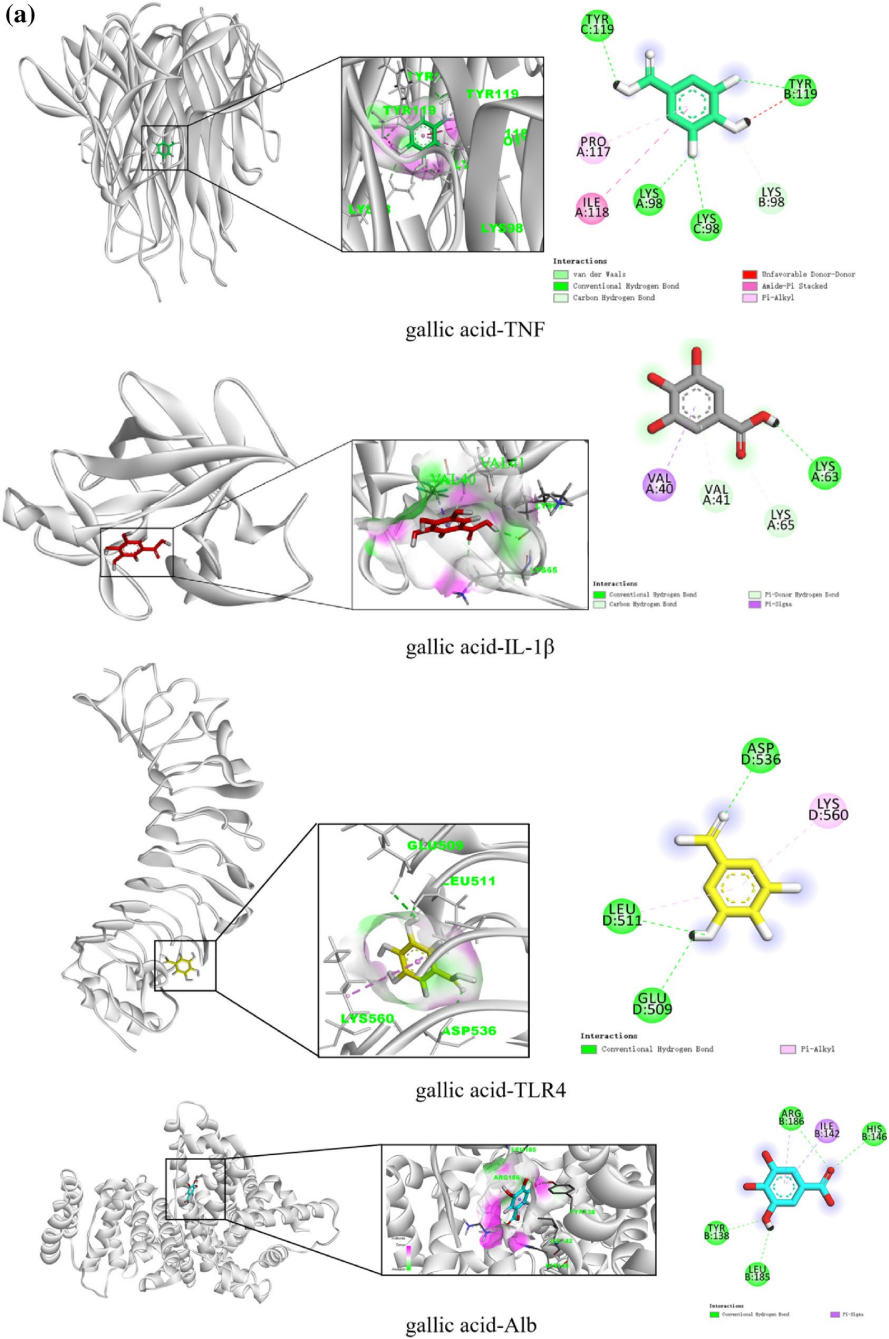

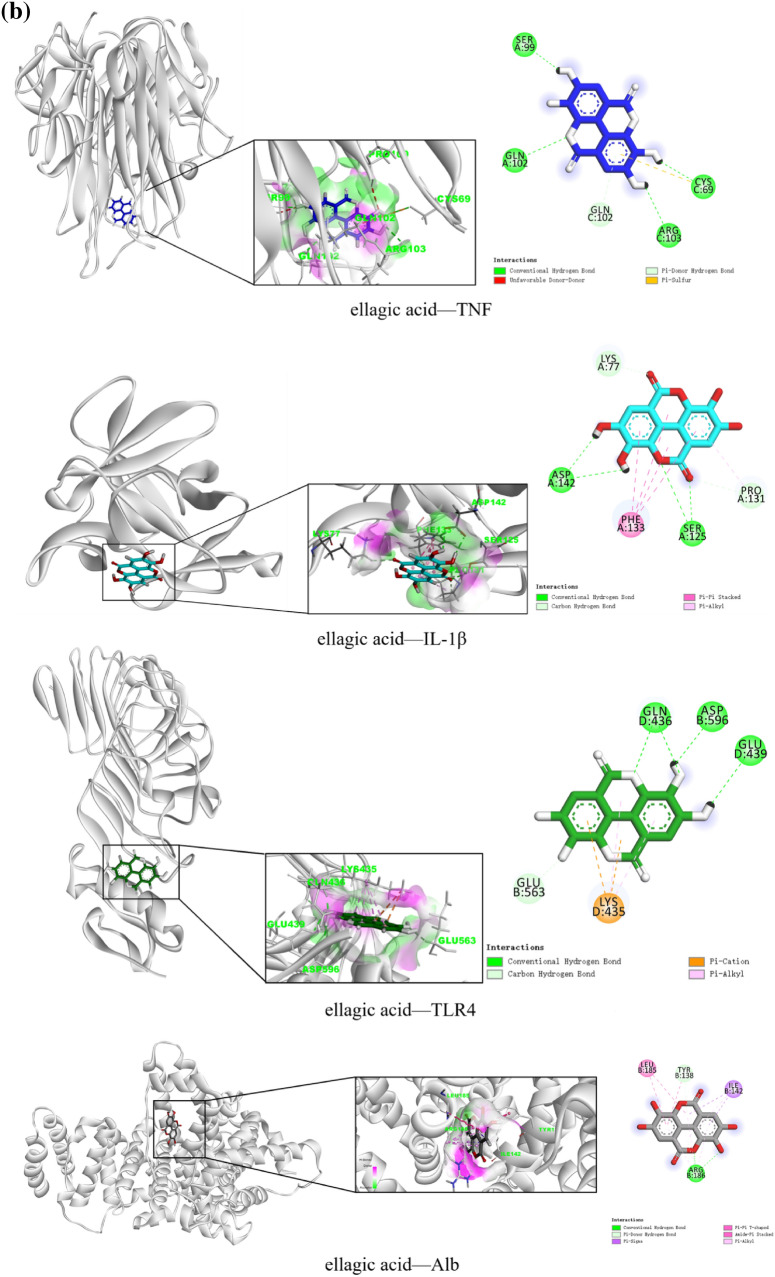

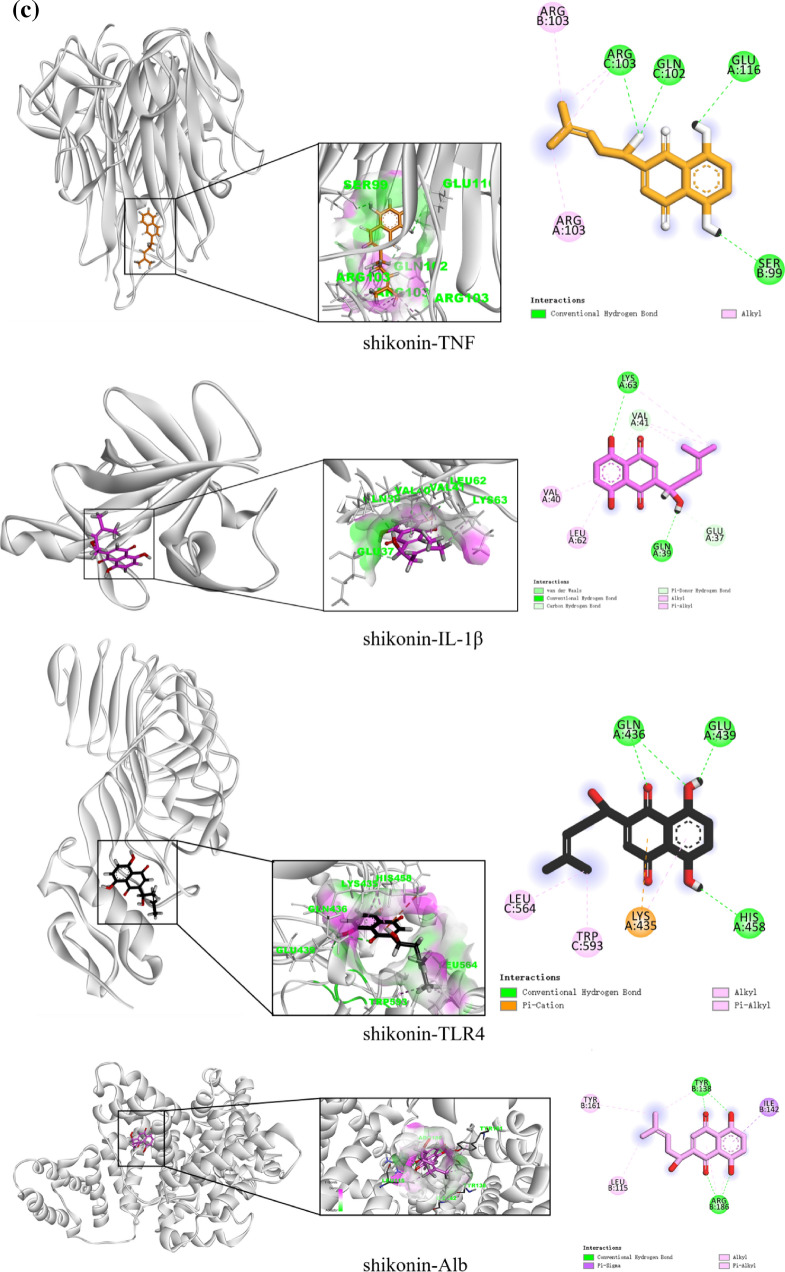
Diagram of molecular docking. **a** 3D ligand interaction diagrams for docking poses of gallic acid in the active sites of TNF, IL-1β, TLR4, and Alb; **b** 3D ligand interaction diagrams for docking poses of ellagic acid in the active sites of TNF, IL-1β, TLR4, and Alb; **c** 3D ligand interaction diagrams for docking poses of shikonin in the active sites of TNF, IL-1β, TLR4, and Alb

The top three core components by degree value in the “component-target-pathway” network are molecularly docked with the top four core targets by degree value in the PPI network. An affinity < 0 means that the ligand and the receptor can bind spontaneously, and the lower the affinity, the stronger the binding effect. An affinity < –5.0 kcal/mol indicates good binding activity. The molecular docking affinity of gallic acid, ellagic acid, shikonin, and TNF, IL-1β, TLR4, and Alb was < –5.0 kcal/mol, demonstrating that the core components and core targets can bind spontaneously and form a relatively stable structure (Table 4). The molecular docking structure is shown in (Fig. 7).

**Table 4:** Results of molecular docking between core components and targets

Traditional Chinese medicine	MolId	MolName	Target	PDB	Affinity (kcal/mol)	Center			Size		
						x	y	z	x	y	z
Turkish gall	MOL000513	Gallic acid	TNF	6RMJ	− 6.8	− 1.641	65.631	127.508	40	40	40
			IL1β	5I1B	− 5.2	11.308	13.79	2.447	62	40	40
			TLR4	2Z66	− 6.8	− 25.107	− 20.278	27.945	46	60	126
			Alb	1AO6	− 6.1	28.423	8.392	23.263	40	46	46
Turkish gall	MOL001002	Ellagic acid	TNF	6RMJ	− 9.1	− 1.641	65.631	127.508	40	40	40
			IL1β	5I1B	− 7.0	11.308	13.79	2.447	62	40	40
			TLR4	2Z66	− 8.7	− 25.107	− 20.278	27.945	46	60	126
			Alb	1AO6	− 8.5	28.423	8.392	23.263	40	46	46
Comfrey	C016101	Shikonin	TNF	6RMJ	− 7.8	− 1.641	65.631	127.508	40	40	40
			IL1β	5I1B	− 7.1	11.308	13.79	2.447	62	40	40
			TLR4	2Z66	− 8.9	− 25.107	− 20.278	27.945	46	60	126
			Alb	1AO6	− 8.5	28.423	8.392	23.263	40	46	46

### Animal experiment

#### Skin apparent index score of each group

**Fig. 8:**
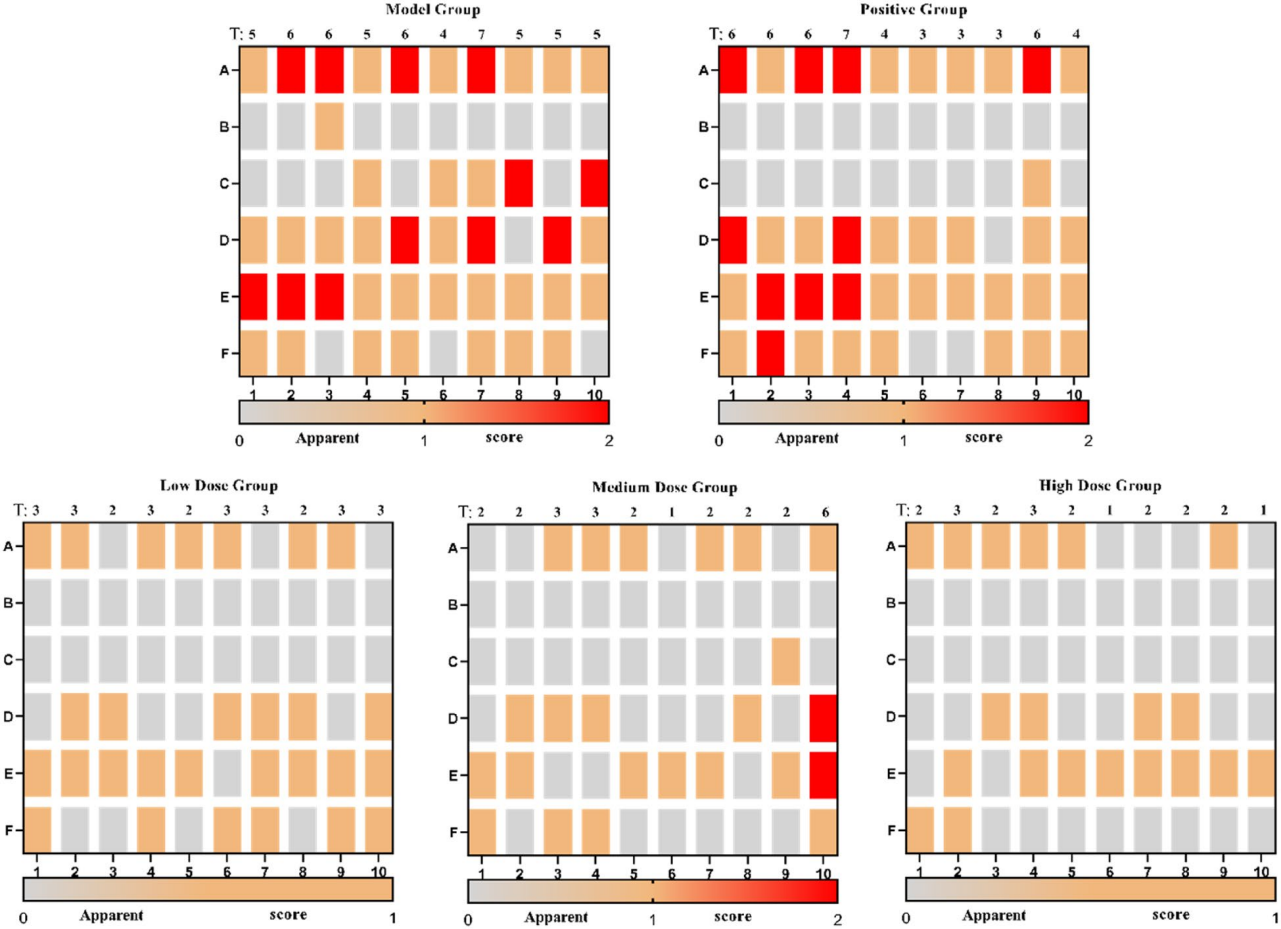
The vertical axis of the picture represents symptoms, **A** Erythema, **B** Edema, **C** Exudation, **D** Desquamation, **E** Lichenification, **F** Dryness. The horizontal axis at the bottom of the picture represents the animal number, 1-10, respectively. The abscissa at the top of the picture represents the total score of the corresponding animal'sapparent index. Gray represents 0 points, yellow represents 1 point, and red represents 2 points

After 14 days of administration, the back skin of rats in the normal group was pale red, delicate, and tender, with a soft texture. The back skin lesions of the rats in the model group were obvious, with skin erythema, infiltration, scales, scabs, rough and thickening texture, pigmentation, and scratches, which still met the diagnostic criteria for chronic eczema. Regarding the observation record of apparent indicators, as shown in Fig. 8, compared to the model group, the erythema, infiltration, scales, and scabs on the back of rats in each administration group were significantly reduced or subsided. Compared to the normal group, the apparent score of rats in the model group was significantly increased (*p* < 0.01). Compared to the model group, the apparent scores in each administration group were significantly decreased (*p* < 0.01), as shown in Table 5.

**Table 5:** Apparent score of zone B (excitation zone) in each group

Grouping	Score
Normal group	0
Model group	5.4 ± 0.8^##^
Positive (0.75 mg/g) group	3.4 ± 0.8**
CTGO low-dose (0.035 g/g) group	2.8 ± 0.4**
CTGO medium-dose (0.07 g/g) group	2.5 ± 1.4**
CTGO high-dose (0.14 g/g) group	2.0 ± 0.7**

^#^*p* < 0.05, ^##^*p* < 0.01 Compared with normal group

^*^p < 0.05, ^**^p < 0.01 Compared with model group

#### HE staining of the skin tissue of rats in each group

**Fig. 9:**
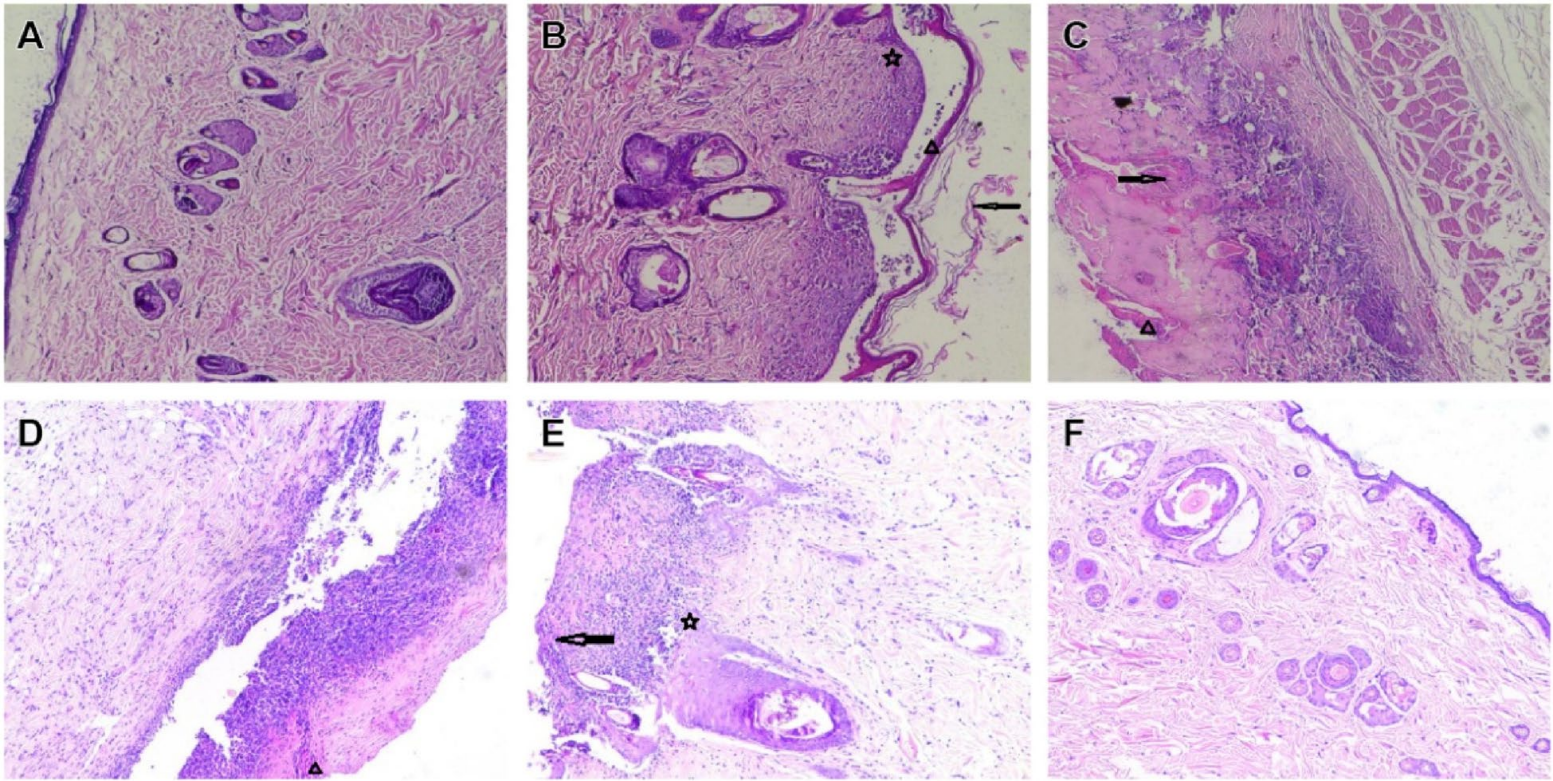
Dermatological observation of rats in each group (HE, × 100). **A** Normal group; **B** model group; **C** positive group; **D** CTGO low-dose group; **E** CTGO middle-dose group; and **F** CTGO high-dose group. Triangle: Hyperkeratosis, arrowhead: Parakeratosis, pentagram: Hypertrophy

**Fig. 10:**
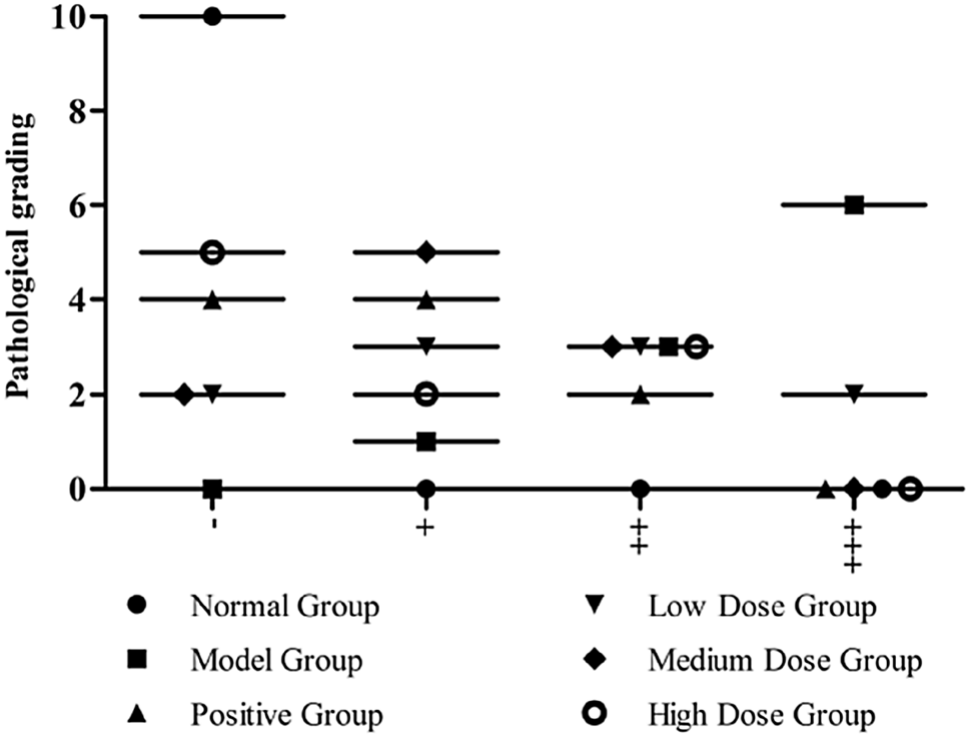
Pathological examination grading (n = 10). The ordinate represents the number of animals, and the abscissa represents the skin lesion degree. Pathological description: –, normal skin tissue, intact tissue structure; + , no obvious change in epidermis, a small amount of inflammatory cell infiltration; +  + , epidermis\dermal cell edema, vast inflammatory cell infiltration; +  +  + , epidermis\dermal numerous inflammatory cell infiltration

The thickness of the cuticle, epidermis, and dermis in the normal group was normal. Model group rats showed chronic eczema, including hyperkeratosis, parakeratosis, hypertrophy of the granular layer, hypertrophy of the spinous layer, and infiltration of numerous inflammatory cells in the epidermis/dermis. After 14 days of administration, hyperkeratosis, parakeratosis, hypertrophy of the granular layer, and hypertrophy of the spinous layer were inhibited in each administration group, and the infiltration of inflammatory cells in the epidermis/dermis was reduced. The results are shown in (Fig. 9). Among them, the number of animals with skin lesion degree “ +  +  + , numerous inflammatory cell infiltration in epidermis/dermis” in the model group was 5, and the numbers of animals in the positive group, low dose group, medium dose group, and high dose group were 0, 2, 0, and 0, respectively. Thus, the medium and high dose groups showed delayed progression of the eczema skin lesions and improved symptoms of skin lesions. The skin lesions were mainly controlled in the absence of obvious changes in the epidermis and a small amount of inflammatory cell infiltration. The results are presented in Fig. 10.

#### Serum IFN-γ and IL-4 levels of rats in each group

**Fig. 11:**
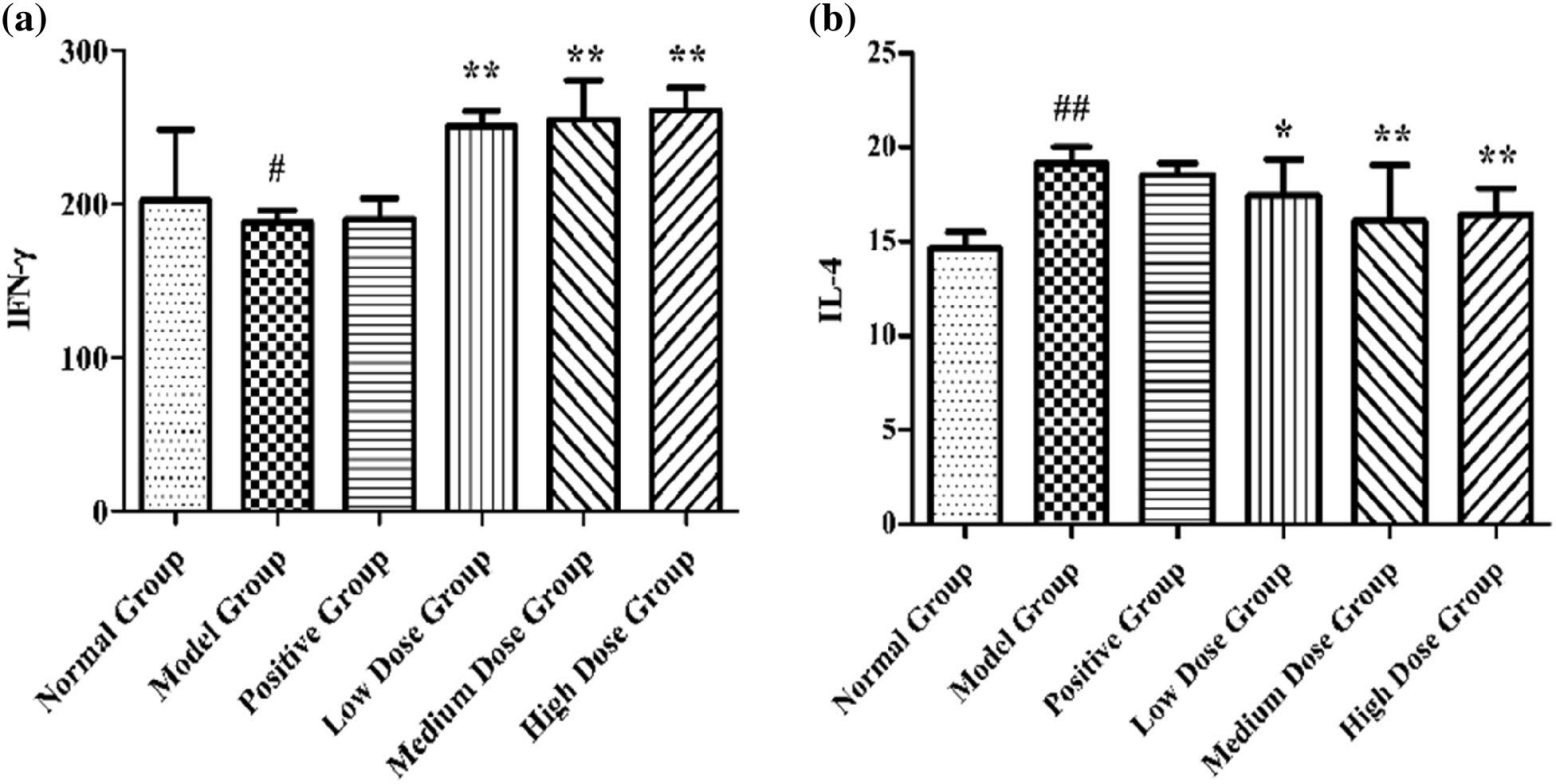
Detection of serum indices in rats (x ± s, n = 10). (a) INF-γ, (b) IL-4. Compared to the normal group ^#^*p* < 0.05; compared to the model group *p < 0.05, **p < 0.01

Compared to the normal group, the levels of IFN-γ in the model group were significantly decreased (^#^*p* < 0.05) and the levels of IL-4 in the model group were significantly increased (^#^*p* < 0.05). Compared to the model group, the level of IFN-γ in the CTGO group was significantly increased (**p < 0.01) and that of IL-4 was significantly decreased (*p < 0.05, **p < 0.01) (Fig. 11). Compared to the model group, the level of IL-4 in the medium and high dose groups decreased significantly (**p < 0.01), and the level of IL-4 in the low dose group decreased significantly (*p < 0.05).

#### Levels of TLR4, NF-κB, IL-1β, and TNFα in the skin tissue of rats in each group

**Fig. 12:**
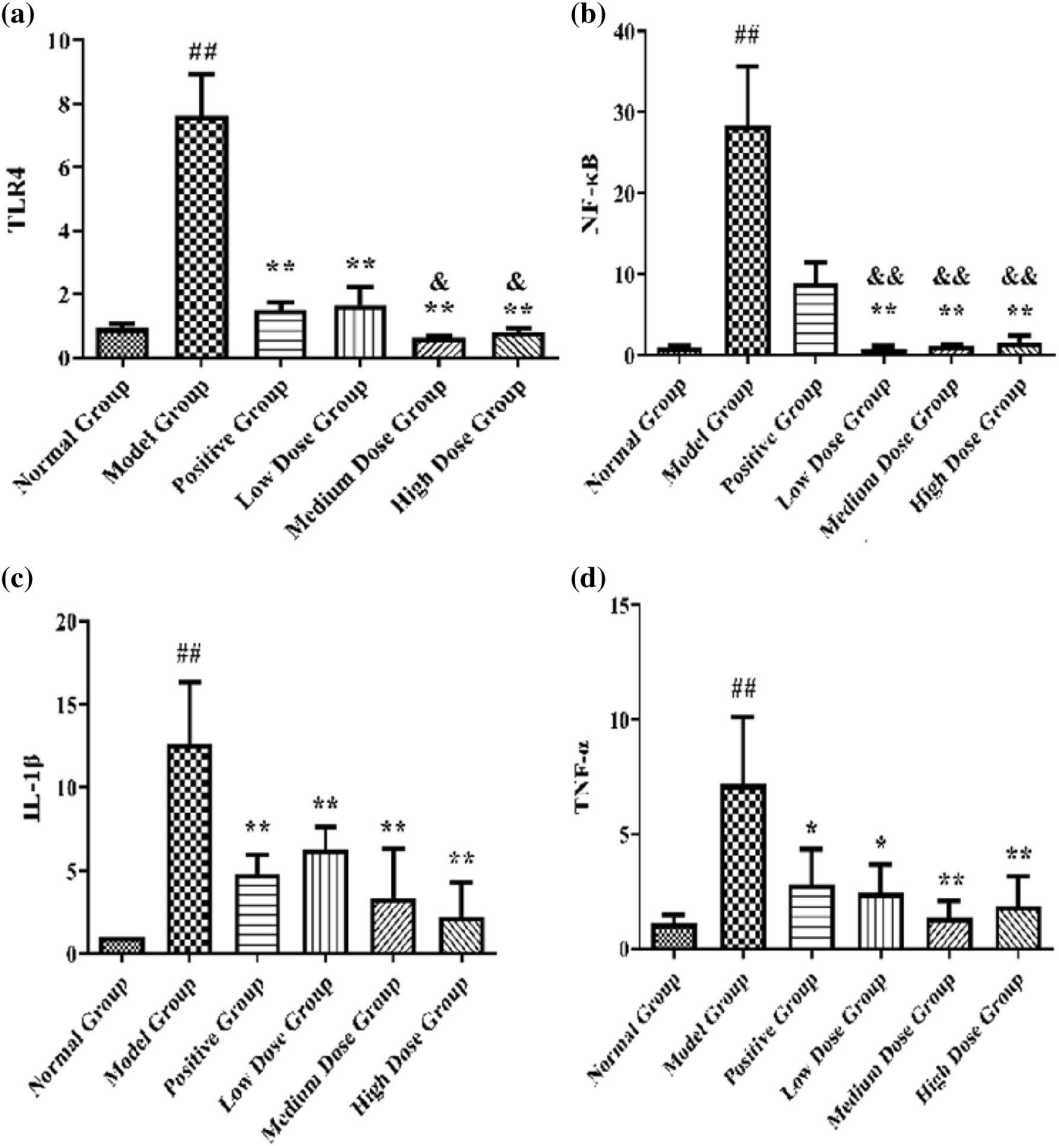
mRNA expression levels in the skin tissue of each group of rats. (a) TLR4, (b) NF-κB, (c) IL-1β, (d) TNFα. Compared to the normal group ^##^*p* < 0.01; compared to the model group *p < 0.05, **p < 0.01; compared to the positive group^**&**^*p* < 0.05, ^**&&**^*p* < 0.01

Compared to the normal group, the contents of TLR4, NF-κB, IL-1β, and TNFα in the skin tissue of rats in the model group were significantly increased (^##^*p* < 0.01, ^###^*p* < 0.001, or ^####^*p* < 0.0001). Compared to the model group, the contents of IL-1β and TNFα in the skin tissue of rats in the positive group and CTGO low-, medium-, and high-dose groups were significantly decreased (*p < 0.05, **p < 0.01, and ***p < 0.001, respectively), and the contents of TLR4 and NF-κB in the skin tissue of rats in the CTGO low-, medium-, and high-dose groups were significantly decreased (**p < 0.01, ***p < 0.001, and ****p < 0.0001, respectively). Compared to the positive group, the contents of TLR4 and NF-κB in the skin tissue of rats in the medium- and high-dose CTGO groups were significantly decreased (^**&**^*p* < 0.05 and ^**&&**^*p* < 0.01, respectively) (Fig. 12).

## Discussion

TCM compounds have multi-component and multi-target synergistic effects, and have been widely used in clinical practice because of their clear curative effect and minor side effects. CTGO has shown good clinical efficacy in the treatment of eczema, but its mechanism of action remains unclear. Based on network pharmacological analysis, we investigated the pharmacodynamics and action mechanism of CTGO in a rat model of eczema.

In this study, a combined strategy of phytochemistry, network analysis, molecular docking, and basic experiments was used to assess the active constituents and potential molecular mechanisms of CTGO on eczema. First, the chemical composition of CTGO was characterized by UHPLC-Q-Orbitrap HRMS for the first time. A total of 29 constituents, mainly belonging to polyphenols, naphthalene quinones, and anthraquinones, were identified. Due to the small number of chemical components identified by mass spectrometry, no more components were identified. To make the results of network pharmacological prediction and analysis more comprehensive, we performed a database and literature search of the chemical constituents of gallic gall and Comfrey, and retrieved 57 chemical constituents. The components identified by mass spectrometry were combined with the retrieved components, and after removing duplicates, 77 components were finally obtained and used as a basis for network pharmacological analysis.

The component-target-pathway network contained 38 active ingredients. The common active components of gallic gall and Comfrey are palmitic acid and β-sitosterol. Their common targets are the core targets of TNF and IL-1β, which rank highly in degree value. The top two components in degree value were gallic acid and ellagic acid, which are the main components of gallic gall, and have 11 common targets, namely, TNF, TGFβ1, RELA, PTGS1, PTGS2, NOS2, MMP9, IL-1β, CYP1B1, CYP1A1, and Alb. All of the targets are closely related to inflammatory, immune, and metabolic reactions. Shikonin is the main chemical component of Comfrey; it can act on nine eczema-related targets, among which TNF and PTGS2 are the common targets of shikonin, gallic acid, and ellagic acid. Gallic acid has a good antibacterial effect, and can effectively inhibit *Staphylococcus aureus*, *Pseudomonas aeruginosa*, *Candida albicans*, and *E. coli* [13]. Gallic acid is also a powerful anti-inflammatory agent that inhibits the secretion of pro-inflammatory cytokines, TNF, IL-6, and IL-1, and indirectly inhibits lipopolysaccharide (LPS) in the cell wall of Gram-negative bacteria to induce an inflammatory response [14, 15]. Ellagic acid is a polyphenolic dilactone, which is a dimer derivative of gallic acid, and it has a variety of biological effects, including antioxidant, anti-inflammatory, anti-proliferative, and antiviral effects [16]. Studies have shown that ellagic acid can reduce leukocyte infiltration and inhibit cell recruitment and the expression of cytokines (IL-4, IL-5, and IL-13) in a time- and dose-dependent manner [17]. Shikonin is the main chemical component of Comfrey, which has anti-inflammatory, anti-tumor, bacteriostatic, liver protection, and immune regulatory effects [18]. Studies have shown that shikonin may inhibit the expression of SP, NK-1R, and ICAM-1 in the skin tissue of eczema model mice, inhibit mast cell degranulation, and alleviate the symptoms of eczema inflammatory injury [19].

The PPI network contains 58 core targets for the treatment of eczema, reflecting the multi-target characteristics of CTGO in the treatment of eczema. TNF-α, IL1β, and IL-4 are important cytokines in the process of skin immunity and inflammation. Under the action of allergens in the skin system, numerous inflammation-mediated factors, such as TNF-α, IL1β, and IL-4, are secreted during epidermal keratinization, all of which interact with each other in the pathogenesis of eczema, thus influencing the development and prognosis of the disease [20, 21]. TLR4 is an important pattern recognition receptor that promotes the expression of TNF-α and IL1β by activating NF-κB [22]. The TLR4 896A > G loci polymorphism is closely associated with disease severity in patients with eczema [23].

Our molecular docking results showed that the overall scores of TNF, TLR4, IL1β, and Alb when docking with four key components were all lower than –5.0 kcal/mol, which suggested that they may play an essential role in CTGO against eczema.

The 38 active ingredients act on 58 targets related to eczema; all of these 58 targets are connected with 118 pathways closely related to inflammation and immunity. All of the above components, targets, and pathways form a network, which may be the mechanism by which CTGO functions to treat eczema. The toll-like and NF-κB pathways are among the first 30 pathways significantly associated with CTGO in the treatment of eczema and are closely related to inflammatory responses. The toll-like pathway affects the NF-κB pathway through regulating NF-κB, and the NF-κB pathway then regulates the downstream of the toll-like pathway. As NF-κB connects the two pathways, they are closely related and mutually regulated. Eight of the 58 intersection targets were enriched on the Toll-like pathway, namely TNF, IL1B, TLR4, RELA, CD14, MAP2K1, RAC1, and IRF3. Seven of the 58 intersection targets were enriched in the NF-κB pathway, namely TNF, IL1B, TLR4, RELA, CD14, PTGS2, and PLAU. PPI topology analysis yielded four core targets, namely TNF, IL1β, TLR4, and Alb, of which TNF, IL1β, and TLR4 were key upstream and downstream targets involved in both of the above pathways. Therefore, we speculate that the toll-like and NF-κB signaling pathways may be the main way by which CTGO functions in the treatment of eczema. The TLR4/NF-κB signaling pathway was selected for validation, and TLR4, NF-κB, TNF-α, and IL1β were finally selected as indicators to be validated in this pathway.

Toll receptors are a class of highly conserved pattern recognition receptors. TLR4 recognizes various ligands of microorganisms and undergoes dimerization upon ligation. MyD88 and non-MyD88 pathways activate mitogen-bound protein kinase, activate NF-κB, upregulate inflammatory cytokines and chemokines, and induce inflammation. These pathways play an important role in the pathogenesis of eczema [24, 25]. NF-κB is a nucleoprotein factor that exists widely in eukaryotic cells, and whose pathological activation is involved in the occurrence and development of various inflammatory diseases, including eczema and asthma. When cells are exposed to external stimulation, the complex of NF-κB and its inhibitory protein IκB are activated, and IκB is phosphorylated, ubiquitinated, and degraded. The resulting free NF-κB is transferred to the nucleus, and the target genes are activated. Inflammatory factors, such as IL-1β and TNF-α, are highly expressed, which can induce the expression of various genes and regulate the inflammatory response. These inflammatory factors are involved in the occurrence and development of related diseases, including eczema and asthma [26–28].

In this study, the rat eczema model was induced by DNCB. When the body is re-exposed to the same antigen after being stimulated by DNCB through the skin, a type IV allergic reaction characterized by skin damage occurs. Repeated stimulation can produce clinical manifestations similar to eczema, and the coincidence degree with the main symptoms of eczema in Chinese medicine is > 70%. Following establishment of the model, many indicators can be used to judge whether the model has been established successfully and whether the drug has a therapeutic effect after administration. Among them, the apparent indexes belong to the core indexes, such as the skin lesion area, itching condition, fur gloss, and skin erythema and edema. Pathological indexes are directly related indexes, and local histopathological changes of skin are reliable indexes to evaluate the degree of skin lesions. Biochemical indexes are indirectly related indexes, and IL-4 and other biochemical indexes will increase after the animal eczema model has been successfully established [29]. After CTGO treatment, the eczema apparent index in the skin area of the lesion was significantly reduced, histopathological manifestations were significantly improved, and the expression level of IL-4 in serum was significantly reduced, indicating that CTGO had a good therapeutic effect on the eczema in rats.

Immune imbalance, especially Th1/Th2 imbalance, plays an important role in the pathogenesis of eczema. IFN-γ is mainly secreted by Th1 cells and can inhibit IL-4 secretion by Th2 cells, as well as inhibit IgE production by B cells. IL-4 is mainly secreted by Th2 cells and mainly inhibits Th1 cell function, and promotes IgE production by B cells, which inhibit and antagonize each other and maintain the Th1/Th2 balance. The expression levels of IFN-γ and IL-4 in normal subjects maintained a relatively balanced state, which is lost in patients with eczema. The experimental results showed that CTGO could significantly reduce IL-4 levels and increase IFN-γ levels, suggesting that CTGO may play a role in the treatment of eczema by promoting Th1/Th2 homeostasis by down-regulating IL-4 levels and up-regulating IFN-γ levels [30, 31].

Patients with eczema have a severe inflammatory response, and many inflammatory factors play a key role in the occurrence and development of eczema. The TLR4/NF-κB signaling pathway is involved in the inflammatory response, where TLR4 recognizes endogenous ligands and is activated via identifying related ligands, and the signal conduction through the MyD88 pathway leads to the activation of NF-κB. This signaling ultimately results in the production of a large amount of IL-1β, TNF-α, and other inflammatory factors, which promote cell proliferation and repair. However, with the excessive activation of NF-κB comes excessive release of these inflammatory factors; then, the responsive activation of TLR4 forms a vicious cycle, which leads to uncontrollable inflammatory response. Consequently, the epidermis produces excessive inflammatory reactions, and the tissue organs are damaged, which result in the occurrence and development of eczema. The results of the animal experiments indicated that CTGO could significantly reduce the expression of IL-1β, TNFα, TLR4, and NF-κB. These changes in key genes suggest that the mechanism of CTGO in treating eczema may be related to the effect of the TLR4/NF-κB signaling pathway [32, 33].

## Conclusions

In conclusion, our experiments showed that CTGO could reduce the expression of IL-1β, TNFα, TLR4, and NF-κB, and significantly promote the expression of IFN-γ, which is consistent with the results of the prediction of network pharmacology and molecular docking. The mechanism of CTGO in treating eczema may be related to regulating related inflammatory factors, promoting the Th1 and Th2 immune balance, and affecting the TLR4/NF-κB signaling pathway.

## Data Availability

The datasets used and analyzed during the current study are available from the corresponding author on reasonable request.

## Abbreviations

CTGO: Compound Turkish gall ointment
UHPLC-Q-Orbitrap HRMS: Ultra-high performance liquid chromatography-Q exactive hybrid quadrupole-orbitrap high-resolution accurate mass spectrometry
SD: Sprague–Dawley
HE: Hematoxylin–eosin
IFN-γ: Interferon-γ
IL-4: Interleukin-4
TLR4: Toll-like receptor 4
NF-κB p65: Nuclear factor kappa-B p65
IL-1β: Interleukin-1β
TNF-α: Tumor necrosis factor-α
RT-qPCR: Real-time quantitative polymerase chain reaction
TCM: Traditional Chinese medicine
DNCB: 2: 4-Dinitrochlorobenzene
PPI: Protein–protein interaction
GO: Gene Ontology
KEGG: Kyoto Encyclopedia of Genes and Genomes
Alb: Albumin
